# Demographic Characteristics and Status of Vaccinated Individuals with a History of COVID-19 Infection Pre- or Post-Vaccination: A Descriptive Study of a Nationally Representative Sample in Saudi Arabia

**DOI:** 10.3390/vaccines10020323

**Published:** 2022-02-18

**Authors:** Yazed AlRuthia, Haya F. Al-Salloum, Omar A. Almohammed, Amani S. Alqahtani, Hana A. Al-Abdulkarim, Yousef M. Alsofayan, Sami S. Almudarra, Sara H. AlQahtani, Abdullah Almutlaq, Khaled Alabdulkareem, Bander Balkhi, Hamoud T. Almutairi, Abdullah S. Alanazi, Yousif A. Asiri

**Affiliations:** 1 Department of Clinical Pharmacy, College of Pharmacy, King Saud University, Riyadh 11451, Saudi Arabia; oalmohammed@ksu.edu.sa (O.A.A.); bbalkhi@ksu.edu.sa (B.B.); halmotairi@KSU.EDU.SA (H.T.A.); yasiri@ksu.edu.sa (Y.A.A.); 2 Pharmacoeconomics Research Unit, Department of Clinical Pharmacy, College of Pharmacy, King Saud University, Riyadh 11451, Saudi Arabia; 3 Department of Pharmacy, King Khalid University Hospital, King Saud University Medical City, Riyadh 12372, Saudi Arabia; hsalloum@ksu.edu.sa; 4 Saudi Food and Drug Authority, Riyadh 13513, Saudi Arabia; as.qahtani@sfda.gov.sa; 5 Drug Policy and Economic Center, National Guard Health Affairs, Riyadh 14812, Saudi Arabia; alabdulkarimha@NGHA.MED.SA; 6 Executive Directorate of Medical Affairs, Saudi Red Crescent Authority, Riyadh 11129, Saudi Arabia; yalsofayan@srca.org.sa; 7 Gulf Center for Disease Prevention and Control, Gulf Health Council, Riyadh 12511, Saudi Arabia; s.almudarra@gulfcdc.org; 8 Department of Public Health, Ministry of Health, Riyadh 11176, Saudi Arabia; sarahalqahtani@moh.gov.sa; 9 Rheumatology Division, Department of Medicine, King Faisal Specialist Hospital and Research Centre, Riyadh 11564, Saudi Arabia; aalmutlaq@kfshrc.edu.sa; 10 Assisting Deputyship for Primary Health Care, Ministry of Health, Riyadh 11176, Saudi Arabia; khalabdulkarim@moh.gov.sa; 11 Department of Clinical Pharmacy, College of Pharmacy, Jouf University, Skaka 42421, Saudi Arabia; asdalananzi@ju.edu.sa

**Keywords:** COVID-19, Oxford–AstraZeneca COVID-19 vaccine, Pfizer COVID-19 vaccine, demographics, Saudi Arabia

## Abstract

Background: Saudi Arabia expedited the approval of some COVID-19 vaccines and launched mass vaccination campaigns. The aim of this study was to describe the demographics of vaccinated COVID-19 cases and compare the mortality rates of COVID-19 cases who were infected post-vaccination in Saudi Arabia. Methods: This was a retrospective cohort study. We retrieved data for COVID-19 cases who were infected pre- or post-vaccination and had received at least one injection of the Oxford–AstraZeneca or Pfizer–BioNTech vaccine from 4 December 2020 to 15 October 2021. Results: The number of patients who were infected and had received at least one dose of a COVID-19 vaccine was 281,744. Approximately 45% of subjects were infected post-vaccination, and 75% of subjects had received the Pfizer–BioNTech vaccine. Only 0.342% of the patients who were infected post-vaccination died, and 447 patients were admitted to ICUs. Most of the patients who were infected with COVID-19 post-vaccination and were admitted to ICUs (69.84%) had received only one dose of the vaccine (*p* < 0.0001). The mean time to infection for patients who had received one and two doses of the Oxford–AstraZeneca vaccine were 27 and 8 days longer than their counterparts who had received one and two doses of Pfizer–BioNTech vaccine, respectively. No difference in the odds of mortality between the Pfizer–BioNTech and Oxford–AstraZeneca vaccines was found (OR = 1.121, 95% CI = [0.907–1.386], *p*-value = 0.291). Patients who had received two doses of the vaccine had significantly lower odds of mortality compared to those who had received one dose (*p* < 0.0001). Conclusions: Vaccines are vital in combating the COVID-19 pandemic. The results of this study show no difference between the Pfizer–BioNTech and Oxford–AstraZeneca vaccines in the rate of mortality. However, the number of vaccine doses was significantly associated with a lower risk of mortality. Future studies should examine the effectiveness of different COVID-19 vaccines using real-world data and more robust designs.

## 1. Introduction

In November 2019, an extremely contagious coronavirus disease was detected in Wuhan City, China, and within a few weeks, it reached several countries around the world. On 11 March 2020, the World Health Organization (WHO) declared the novel coronavirus disease (COVID-19) outbreak as a pandemic [1]. The number of COVID-19 cases kept increasing, reaching approximately 282 million cases and 5.4 million deaths as of 3 December 2022 [2]. In Saudi Arabia, the first COVID-19 case was reported on Monday 2 March 2020 [3]. As of 13 January 2022, the Saudi Ministry of Health (MoH) has reported more than 599,000 cases and 8901 deaths [4]. Around the world, we have witnessed significant variability in the signs, symptoms, risk factors, rates of hospital and intensive care unit (ICU) admissions, utilization rates of mechanical ventilators, and mortality rates [5,6,7]. However, headaches, fever, fatigue, dyspnea, sore throat, joint pain, and diarrhea are the most common symptoms of COVID-19 [3,8,9]. According to the Saudi MoH, the mortality rate in Saudi Arabia is 1.48% [4], which is in line with the globally reported range of fatality rates [7].

To mitigate the high COVID-19 transmission rates, there are several measures that can be implemented, including social distancing, masking, and hand hygiene [10]. In addition, several countries, including Saudi Arabia, have enforced partial and temporary complete lockdowns and travel bans to contain the transmission of the virus at the beginning of the COVID-19 pandemic [11,12]. These measures were taken to mitigate the impact of the pandemic on the already strained healthcare systems in many countries [13,14,15]. In Saudi Arabia, the government utilized the full capacity of its public healthcare system with 80,000 beds and 8000 ICU beds to manage the rising number of COVID-19 cases that required hospitalization. However, the pandemic required building new hospitals and testing facilities, expanding the ICU bed capacity, and obtaining personal protective equipment, hospital equipment, and sanitizing supplies, which had a significant negative impact on the healthcare budget and on the availability of diverse essential medications [16,17]. Therefore, there is a race against time to manufacture and distribute effective vaccines and therapies to reduce the rates of transmission, hospitalization, and mortality [18,19].

As of 14 June 2021, several vaccines have been developed, approved, and marketed for vaccination against COVID-19 among adults (≥18 years) [20]. These COVID-19 vaccines, such as Pfizer–BioNTech (BNT162b2) [21], Moderna (mRNA-1273) [22], Oxford–AstraZeneca (ChAdOx1 nCoV-19) [23], Johnson & Johnson (Ad26.COV2-S) [24], Gamaleya (Sputnik V) [25], Sinopharm (BBIBP-CorV) [26] and Sinovac Biotech (CoronaVac) [26], have variable efficacy in terms of the risk of infection, hospitalization, and mortality [20]. Their rates of efficacy in reducing the rates of infection and symptomatic cases range from 50% for CoronaVac to 95% for Pfizer–BioNTech [20]. Nonetheless, countries that made vaccines available to the public and achieved high vaccination rates have seen a significant decline in the rates of hospitalization and mortality [27,28,29,30]. For example, according to national surveillance data in Israel, individuals vaccinated with Pfizer–BioNTech who received at least one dose and were followed-up for at least 14 days had 73% and 79% lower rates of symptomatic COVID-19 cases and deaths, respectively [27]. In the Italian province of Pescara, the rates of COVID-19 infection were compared among 69,539 vaccinated adults, who received at least one dose of Pfizer–BioNTech, Oxford–AstraZeneca, or Moderna, and 175,687 unvaccinated adults. The rates of infection and mortality for the vaccinated adults were 0.12% and 0.0043%, respectively, in comparison to 4% and 0.14% for their unvaccinated counterparts [28]. Another study examined the effectiveness of CoronaVac in preventing COVID-19 infection, and COVID-19-related hospitalization and death among a cohort of 10.2 million people aged 16 years or older in Chile [29]. Among those partially vaccinated (e.g., received one dose of CoronaVac), the vaccine effectiveness rates for the prevention of COVID-19 infection, hospitalization, ICU admission, and death were 15.5%, 37.4%, 44.7%, and 45.7%, respectively. The vaccine effectiveness rates for the prevention of COVID-19 infection, hospitalization, ICU admission, and death among those fully vaccinated (e.g., received two doses of CoronaVac) were 65.9%, 87.5%, 90.3%, and 86.3%, respectively [29]. Even though different efficacy rates against COVID-19 infection for Pfizer–BioNTech and Oxford–AstraZeneca have been reported in clinical trials for older adults [20], these differences were not observed in a case-control study in England that included more than 156,000 elderly patients aged 70 years or older who were infected between December 2020 and February 2021 [30].

Few studies have examined the effectiveness of COVID-19 vaccines in the Middle East [31,32]. In Qatar, the effectiveness of Pfizer–BioNTech decreased after five to seven months of the second dose, reaching approximately 20%, particularly for the Beta and Delta variants [31]. In contrast, a single dose of either Pfizer–BioNTech or Oxford–AstraZeneca is 92.17% effective in preventing symptomatic COVID-19 infection eight months post-vaccination, according to a single-center study that included 18,543 vaccinated subjects in Saudi Arabia [32].

Saudi Arabia was one of the first countries in the Middle East to approve and procure Pfizer–BioNTech and Oxford–AstraZeneca vaccines and make the vaccination free and mandatory for all adult and teenage (e.g., 12–17 years) citizens and residents [33,34]. In addition, Johnson & Johnson, Moderna, Sinopharm, and Sinovac vaccines were approved in 2021 [35]. Nevertheless, only Pfizer–BioNTech, Oxford–AstraZeneca, and Moderna have been available for mass vaccination campaigns [35]. According to the Saudi MoH, more than 53 million COVID-19 vaccine doses have been administered as of 13 January 2022 [4]. However, there are no studies describing the demographic characteristics of vaccinated subjects in Saudi Arabia who were infected with COVID-19 after the start of the first mass vaccination campaign, which started in December 2020. Moreover, no study has examined the time from vaccination to infection and survival rates among those who were infected post-vaccination. Therefore, the objectives of this study were to (1) describe the demographic characteristics of patients who were infected with COVID-19 during the mass vaccination period, (2) explore the time to infection based on the type of vaccine and age group, and (3) examine the impact of the type of vaccine and number of doses or injections on the survival of those who were infected post-vaccination in Saudi Arabia.

## 2. Methods

### 2.1. Study Design

This was a retrospective descriptive cohort study. Using data from the Saudi Arabian National Vaccination Record, we retrospectively recruited from 4 December 2020 to 15 October 2021 adult (≥18 years) COVID-19 patients who had received a COVID-19 vaccination (Pfizer–BioNTech or Oxford–AstraZeneca) before or after the PCR-confirmed COVID-19 infection. We retrieved the survival status for those who were infected with COVID-19 post-vaccination from the Health Electronic Surveillance Network (HESN) database of the Saudi MoH for COVID-19 patients. Patients who were not vaccinated during this timeframe (4 December 2020 to 15 October 2021) were not included in the analysis. We obtained demographic variables, such as age, sex, and geographic location, and number of injections and type of vaccination (Pfizer–BioNTech or Oxford–AstraZeneca) from the National Vaccination Record. From the HESN database, we obtained data on the type of COVID-19 variant, time from vaccination to infection for those who were infected with COVID-19 after vaccination, whether patients were admitted to ICUs during their PCR-confirmed COVID-19 infection, length of hospital stay (LOS), and their status (e.g., confirmed infection, recovered, or deceased).

### 2.2. Statistical Analysis

We used descriptive statistics including mean, standard deviation, frequencies, and percentages to present the demographic and vaccination status of COVID-19 patients who were infected and received the vaccine from 4 December 2020 to 15 October 2021. We used basic inferential tests, such as Chi-square, Fisher’s exact test, Student’s *t*-test, and one-way ANOVA, to compare the percentages and mean of categorical and numerical variables, respectively, for those who received Pfizer–BioNTech or Oxford–AstraZeneca and those who received one to two injections of the vaccines. We conducted multiple logistic regression analyses to examine the relationship between different variables (type of vaccine, number of injections, ICU admission, age, and sex) and survival status (recovered vs. deceased) for patients who were infected after being vaccinated with either vaccine (Pfizer–BioNTech or Oxford–AstraZeneca) and separately for each cohort based on the vaccine type. We presented the results in tables and figures and conducted all statistical analyses using SAS^®®^ version 9.4 (SAS^®®^ Institute, Cary, NC, USA).

### 2.3. Ethical Approval for the Study Protocol

The Ethics Review Board Committee of the Central MoH (21–92E) in Riyadh, Saudi Arabia, approved our study.

## 3. Results

**Figure 1:**
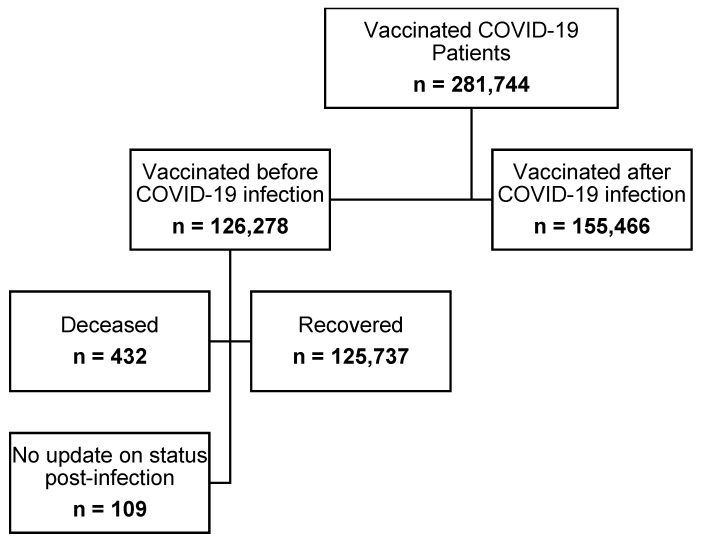
Schematic diagram of sample recruitment.

**Figure 2:**
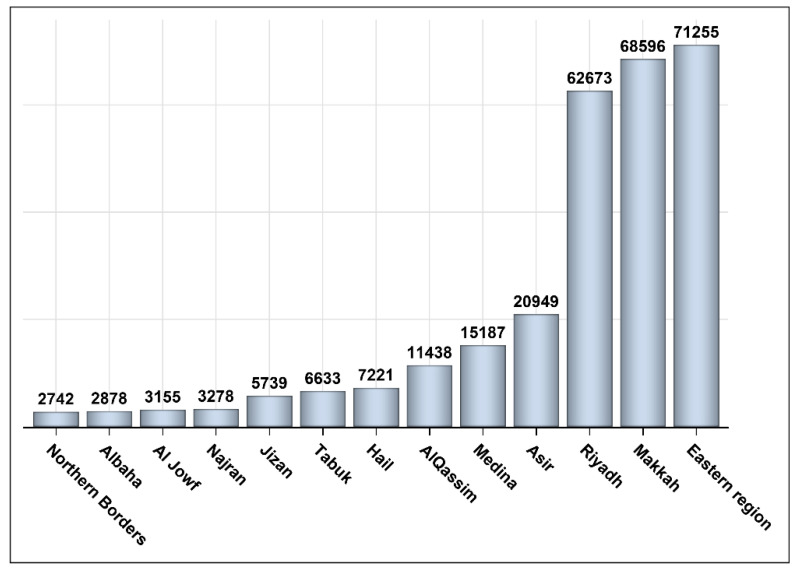
Number of persons vaccinated in each region.

**Figure 3:**
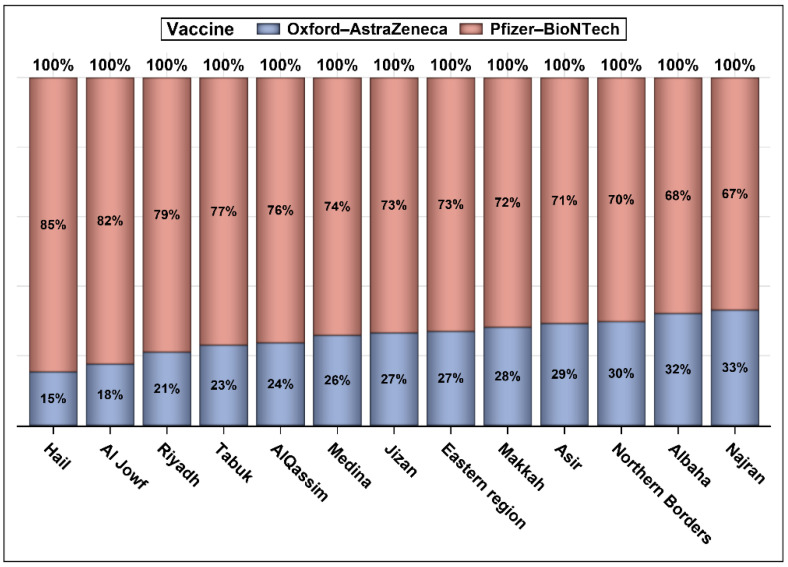
Percentage of persons in each region vaccinated with Pfizer–BioNTech and Oxford–AstraZeneca vaccines.

**Figure 4:**
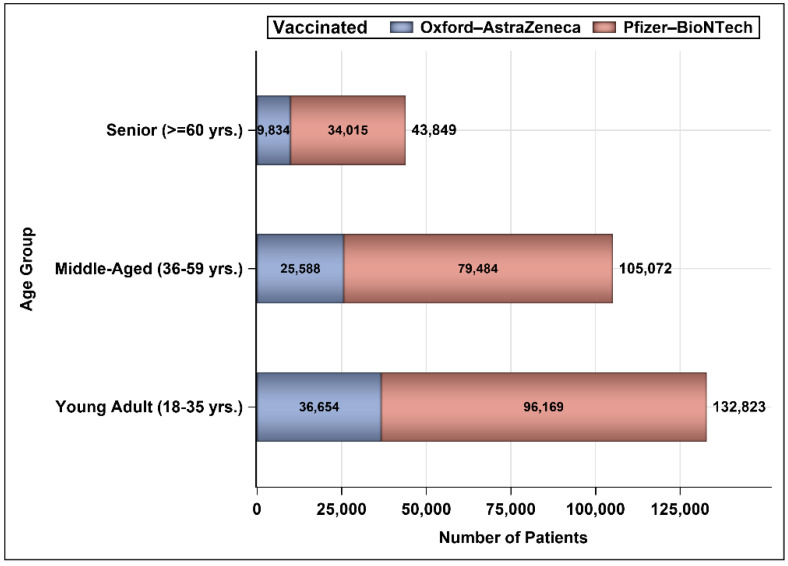
Number of persons based on their age group.

The number of patients who were infected with COVID-19 and were vaccinated between 4 December 2020 and 15 October 2021 was 281,744. Out of the 281,744 vaccinated individuals, 155,466 (55.18%) were vaccinated following recovery and 126,278 (44.82%) were vaccinated prior to COVID-19 infection. Most of those who were infected post-vaccination (99.57%) had recovered; 432 (0.34%) were deceased, and 109 (0.086%) did not have an update on their status (Figure 1). Most of the patients were from the three main regions in the kingdom (Riyadh, Eastern region, and Makkah; Figure 2). The vast majority of patients were vaccinated with the Pfizer–BioNTech COVID-19 vaccine (74.42%); and 25.58% had received the Oxford–AstraZeneca vaccine (Figure 3). We obtained almost the same percentages for the 13 different regions of the kingdom (Figure 3). Approximately 79% of patients who were infected with COVID-19 prior to vaccination and 69% of those vaccinated before the infection were vaccinated with Pfizer–BioNTech. Approximately 52% of patients were female. Most patients who received the Pfizer–BioNTech vaccine (53.69%) were female, while 53.49% of patients who received the Oxford–AstraZeneca vaccine were male. Approximately 0.16% of patients were admitted to ICUs during the COVID-19 infection with no significant differences between the patients according to the vaccine type (Pfizer–BioNTech vs. Oxford–AstraZeneca). Approximately two-thirds of patients vaccinated with Pfizer–BioNTech received two injections and less than 50% of those vaccinated with Oxford–AstraZeneca received two injections (Table 1). Figure 4 shows that ~47% of the patients were young adults (18–35 years), 37% were middle-aged (36–59 years), and 16% were seniors with the majority (>70%) of them being vaccinated with Pfizer–BioNTech.

**Figure 5:**
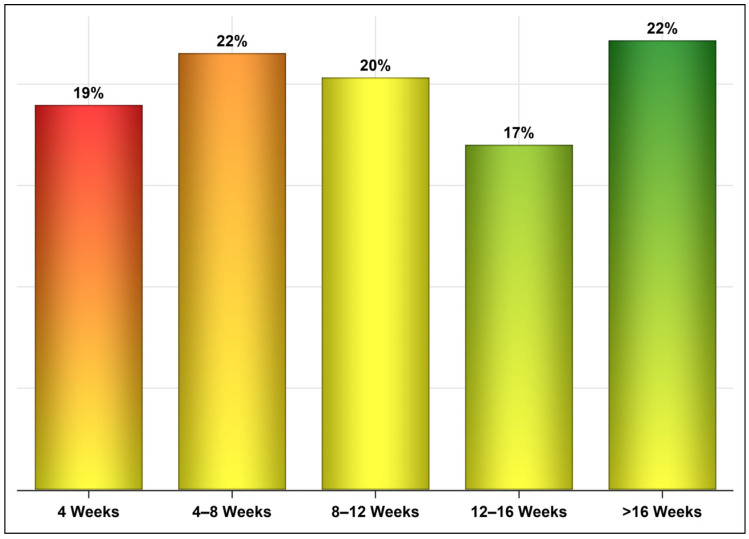
Percentage of patients who were infected with COVID-19 after vaccination across different time intervals. The similar percentages of patients who were infected across different time intervals suggest that the time to infection might be related more to the patient characteristics (e.g., medical conditions and sociodemographics) and behavior (e.g., adherence to social distancing and wearing masks).

**Figure 6:**
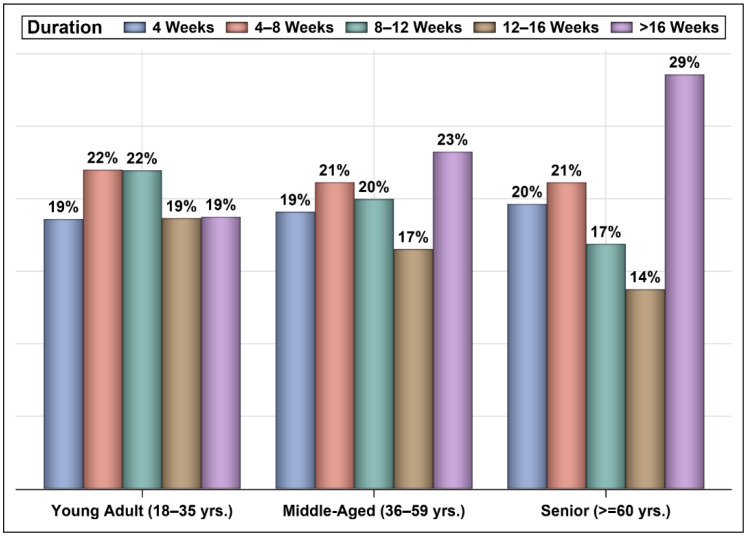
Time from vaccination to infection across age groups (n = 126,169).

**Figure 7:**
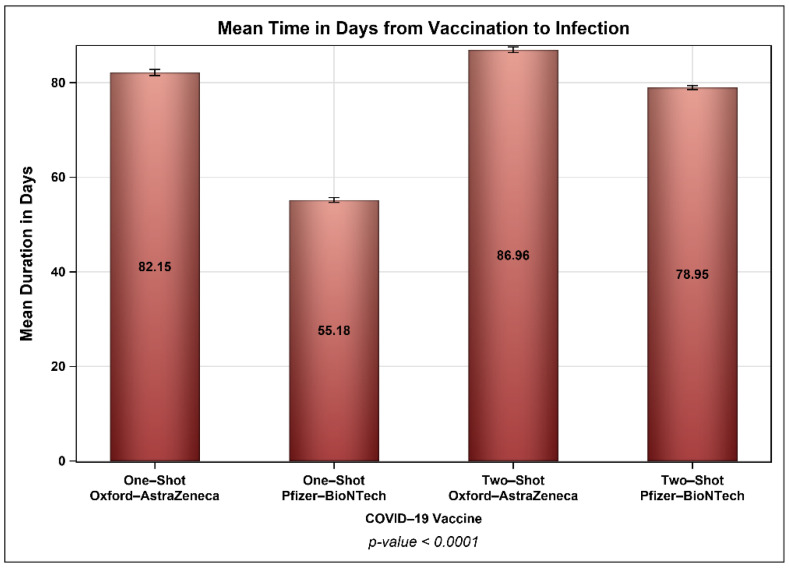
Time to infection based on the vaccine (Pfizer–BioNTech vs. Oxford–AstraZeneca) and number of injections. *p*-value is for one-way ANOVA.

**Figure 8:**
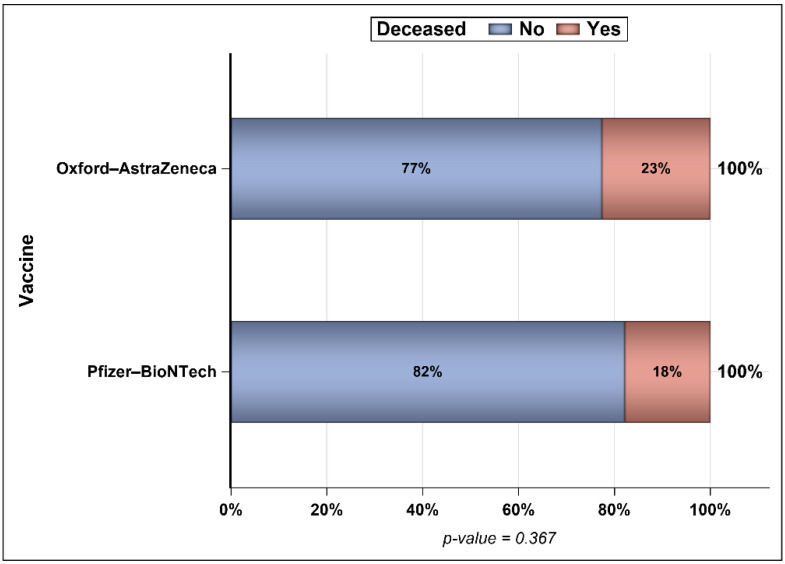
Percentage of deceased patients among those infected after vaccination and admitted to ICUs (n = 252).

**Figure 9:**
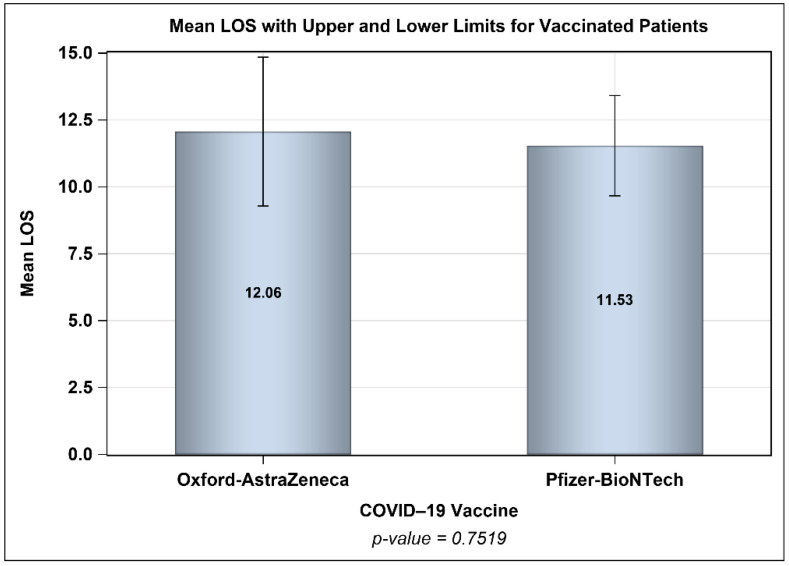
Mean length of stay (LOS) for vaccinated patients (Pfizer–BioNTech vs. Oxford–AstraZeneca; n = 252) prior to infection.

Figure 5 shows the percentages of patients who were infected with COVID-19 after vaccination across different time intervals, and Figure 6 presents the percentages of patients from each age group (young adults, middle-aged, and seniors) who were infected with COVID-19 across different time intervals with no significant difference between the two vaccines. The mean periods from vaccination to infection were 27 and 8 days longer for patients who received one and two doses of the Oxford–AstraZeneca vaccine than for patients who received one and two doses of the Pfizer–BioNTech vaccine, respectively (Figure 7). Even though ~23% of those vaccinated with Oxford–AstraZeneca and admitted to ICUs post-vaccination were deceased in comparison to 18% of those vaccinated with Pfizer–BioNTech, this difference was not significant (Figure 8). Similarly, there were no differences in the LOS between those admitted to ICUs post-vaccination with Oxford–AstraZeneca and those vaccinated with Pfizer–BioNTech (Figure 9). Among those admitted to ICUs, 84 patients (33.33%) received the Oxford–AstraZeneca vaccine, and 168 patients (66.66%) received the Pfizer–BioNTech vaccine. However, 69.84% of those admitted to ICUs had received only one injection of either vaccine (*p* < 0.0001).

The odds of mortality among COVID-19 patients who were infected post-vaccination (n = 126,169) were not significantly higher between those vaccinated with Pfizer–BioNTech and those vaccinated with Oxford–AstraZeneca (OR = 1.121, 95% CI = [0.907–1.386], *p* = 0.291) after controlling for sex, age, age, ICU admission, and number of injections (Table 2). Females had lower odds of mortality compared to males (OR = 0.712, 95% CI = [0.583–0.871], *p* = 0.0009). In addition, older age was associated with higher odds of mortality (OR = 1.099, 95% CI = [1.091–1.106], *p* < 0.0001). ICU admission was associated with more than 15 times higher odds of mortality than no ICU admission (OR = 1.099, 95% CI = [1.091–1.106], *p* < 0.0001). Patients who received two injections of a COVID-19 vaccine regardless of the vaccine type had 85% lower odds of mortality compared to their counterparts who only received one injection (OR = 0.149, 95% CI = [0.118–0.189], *p* < 0.0001). Table 3 and Table 4 show the association between the number of vaccine injections, sex, age, and ICU admission in patients who received Oxford–AstraZeneca and Pfizer–BioNTech vaccines, respectively. There were no significant differences in the association between mortality and number of injections, age, and ICU admission. However, we obtained an association between females and mortality. Specifically, males vaccinated with Oxford–AstraZeneca were not associated with higher odds of mortality.

## 4. Discussion

The COVID-19 pandemic has posed serious health, economic, educational, and social problems globally [36]. Multiple countries continue to struggle to contain this pandemic using different measures, including vaccination [37]. Even though the stunning pace of COVID-19 vaccines’ development, approval, and rollout has raised some concerns regarding their safety and efficacy [38], they have proven to be highly effective in reducing the incidence of infection and the risk of COVID-19-related hospitalization and death [20,28,29,30]. The WHO has urged countries to prioritize vaccinations, especially of the elderly and those with medical comorbidities, such as diabetes, cardiovascular disease, and cancer, which render them more vulnerable to COVID-19 infection and its severe complications [39,40]. To ensure the global equitable access to these vaccines, different international bodies such as the Global Alliance for Vaccines and Immunizations and some governments of high-income countries have implemented various initiatives that encourage vaccine donations to low- and middle-income countries [41,42]. Saudi Arabia responded rapidly to this pandemic by implementing various measures to lessen its impact, including providing free COVID-19 vaccines to all citizens and residents in the kingdom and donating vaccines to underdeveloped nations [43]. However, no studies have described the demographic characteristics (e.g., age, sex, and region) of vaccinated individuals who were infected after the initiation of the mass vaccination campaigns, the time to infection, the specific vaccine received, and the impact of different COVID-19 vaccines on survival.

Unsurprisingly, most of the COVID-19 cases in this nationally representative sample received the Pfizer–BioNTech vaccine, which was mainly due to supply-chain issues [44,45]. The fact that approximately 126,000 subjects were infected with COVID-19 post-vaccination confirms the findings of many studies that COVID-19 vaccines are effective at reducing the risk of transmission, hospitalization, and mortality, but they do not eliminate it [46]. Moreover, these findings clearly indicate that the effectiveness of vaccines wane over time [31]. Another interesting finding was the difference in the time to infection post-vaccination. Even though most COVID-19 cases (e.g., 59%) were infected eight weeks or later post-vaccination, 41% of the cases were infected in the first eight weeks post-vaccination with no significant differences among the different age groups. The mean time to infection was almost four weeks longer for patients who received one injection of the Oxford–AstraZeneca vaccine than those who received one injection of the Pfizer–BioNTech vaccine. Similarly, the mean time to infection was eight days longer for those who received two injections of the Oxford–AstraZeneca vaccine than for those who received two injections of the Pfizer–BioNTech vaccine. Bernal et al. reported that the effectiveness of a single dose of the Pfizer–BioNTech vaccine in preventing symptomatic COVID-19 disease was 61% from 28–34 days post-vaccination and subsequently plateaued. The effectiveness of a single dose of the Oxford–AstraZeneca vaccine was 60% from 28–34 days post-vaccination and increased to 73% from 35 days onwards [30].

There were only 432 deaths (0.342%) among the 126,169 COVID-19 cases who were infected post-vaccination between 4 December 2020 and 15 October 2021. This number of deaths only represents 15.28% of the total number of deaths reported during this period (n = 2827), and the remaining 2395 deaths were unvaccinated subjects [4]. In addition, more than two-thirds of those admitted to ICUs received one injection of a COVID-19 vaccine (Oxford–AstraZeneca or Pfizer–BioNTech). These findings are in accordance with the published literature that reported the effectiveness of vaccines in reducing mortality and the importance of full immunization against COVID-19, which results in lower rates of hospitalization and mortality [47,48,49]. Even though there was no difference in the odds of mortality between the two vaccines (Oxford–AstraZeneca and Pfizer–BioNTech), each one of these vaccines resulted in significant reduction in the odds of mortality, as previously reported [30]. Although the second dose of both vaccines (Oxford–AstraZeneca and Pfizer–BioNTech) significantly reduced the odds of mortality, the second dose of the Pfizer–BioNTech vaccine reduced the odds of mortality to a greater extent than the second dose of the Oxford–AstraZeneca vaccine. Chemaitelly et al. reported on the waning effectiveness of the Pfizer–BioNTech vaccine after 5–7 months of vaccination and the need for a booster, especially against the Delta variant [31,49]. Male subjects had higher odds of mortality than their female counterparts after controlling for the number of vaccine doses, age, and ICU admission. This is consistent with most published studies, which showed a higher risk of COVID-19 related hospitalization, longer LOS, and mortality among males [50,51]. Additionally, old age was associated with a higher risk of COVID-19-related mortality as previously reported [51]. Patients who were admitted to ICUs were at significantly higher risk of mortality after controlling for age, sex, vaccine type (Oxford–AstraZeneca or Pfizer–BioNTech), and number of vaccine doses. This is not surprising, because most admitted patients to ICUs were in critical condition, had multiple comorbidities, and were most likely placed on mechanical ventilators, which in turn increased their risk of death, as found in multiple studies [16,52]. The study findings contribute to the wealth of knowledge that has been accumulating over the past 12 months, which unequivocally demonstrates the value of COVID-19 vaccines in reducing the rates of hospitalization and mortality [53]. Moreover, these findings help dispel any misinformation about COVID-19 vaccines and encourage those who are still hesitant to vaccinate to receive their vaccine doses as soon as possible [53,54].

To the best of our knowledge, this is the first study that describes the demographic characteristics and status of vaccinated subjects in Saudi Arabia using a nationally representative sample. However, our study had a few limitations. First, the study did not include any information about, nor was it controlled for, patients’ chronic health conditions, such as diabetes, hypertension, dyslipidemia, and cardiovascular disease, which are prevalent among the Saudi population [55,56]. Second, the study did not include information on the patients’ history of hospitalization and only included ICU admission due to the lack of hospitalization history in the database from which the data were retrieved. Third, the study did not control for the type of COVID-19 variant, which can have a significant impact on the results of the study [20,48,49]. Finally, the study did not compare mortality rates among those who were vaccinated post-infection and those who were infected with COVID-19 post-vaccination.

## 5. Conclusions

Our study emphasizes the importance of full immunization in reducing the rates of mortality among the Saudi population. Even though our study had a retrospective design, its findings highlight the importance of full immunization to the public and assist decision makers in assessing the benefit of different vaccines using real-world data. Future studies should examine the effectiveness of different COVID-19 vaccines and booster injections in preventing infections and reducing the rates of hospitalization and mortality after controlling for potential confounders, such as chronic health conditions, using real-world data.

## Figures and Tables

**Table 1:** Persons’ baseline characteristics.

Characteristic	COVID-19 Vaccine		*p*-Value *	Total
	Pfizer–BioNTech N (%)	Oxford–AstraZeneca N (%)		
COVID-19 Infection				
Pre-Vaccination	122,706 (58.52)	32,760 (45.45)	<0.0001	155,466 (55.18)
Post-Vaccination	86,962 (41.48)	39,316 (54.55)		126,278 (44.82)
Gender				
Female	112,575 (53.69)	33,522 (46.51)	<0.0001	146,097 (51.85)
Male	97,093 (46.31)	38,554 (53.49)		135,647 (48.15)
COVID-19 related ICU Admissions				
Yes	331 (0.16)	116 (0.16)	0.858	447 (0.16)
No	209,337 (99.84)	71,960 (99.84)		281,297 (99.84)
Number of injections				
**1**	72,000 (34.34)	38,314 (53.16)	<0.0001	110,314 (39.15)
**2**	137,668 (65.66)	33,762 (46.84)		171,430 (60.85)

* Chi-squared test for categorical variables, Student’s *t*-test for continuous ones.

**Table 2:** Multiple logistic regression for the association between the administration of vaccines (Oxford n = 39,320 vs. Pfizer n = 86,849) and mortality among COVID-19 patients vaccinated prior to infection (n = 126,169).

Variable	Odds Ratio (OR)	*p* Value	95% Confidence Interval
Pfizer–BioNTech vs. Oxford–AstraZeneca	1.121	0.2916	0.907–1.386
Female vs. Male	0.712	0.0009	0.583–0.871
Age	1.099	<0.0001	1.091–1.106
ICU admission	15.746	<0.0001	10.757–23.048
Number of injections (2 injections vs. 1 injection)	0.149	<0.0001	0.118–0.189

**Table 3:** Multiple logistic regression for the association between the number of vaccine injections and mortality among vaccinated COVID-19 patients with Oxford–AstraZeneca vaccine (n = 39,320).

Variable	Odds Ratio (OR)	*p* Value	95% Confidence Interval
Number of injections (2 injections vs. 1 injection)	0.215	<0.0001	0.141–0.328
Female vs. Male	0.880	0.4755	0.620–1.250
Age	1.105	<0.0001	1.092–1.118
ICU admission	19.001	<0.0001	10.125–35.660

**Table 4:** Multiple logistic regression for the association between the number of vaccine injections and mortality among vaccinated COVID-19 patients with Pfizer–BioNTech vaccine (n = 86,849).

Variable	Odds Ratio (OR)	*p* Value	95% Confidence Interval
Number of injections (2 injections vs. 1 injection)	0.127	<0.0001	0.096–0.168
Female vs. Male	0.634	0.0010	0.496–0.810
Age	1.095	<0.0001	1.086–1.104
ICU admission	13.730	<0.0001	8.505–22.164

## Data Availability

The data are available upon request to the corresponding author (Y.A.).
